# microRNAs: the art of silencing in the ear

**DOI:** 10.1002/emmm.201100922

**Published:** 2012-06-29

**Authors:** Anya Rudnicki, Karen B Avraham

**Affiliations:** Sackler Faculty of Medicine, Department of Human Molecular Genetics and Biochemistry, Tel Aviv UniversityTel Aviv, Israel

**Keywords:** auditory, cochlea, deafness, mice, vestibule

## Abstract

MicroRNAs (miRNAs) are small non-coding RNAs that regulate gene expression through the RNA interference (RNAi) pathway and by inhibition of mRNA translation. miRNAs first made their appearance in the auditory and vestibular systems in 2005, with the discovery of a triad of hair cell-specific miRNAs later found to be involved in both human and mouse deafness. Since then, miRNAs have been implicated in other medical conditions related to these systems, such as cholesteatomas, vestibular schwannomas and otitis media. Due to the limitations in studying miRNAs and their targets derived from human inner ears, animal models are vital in this field of research. Therefore their role in inner ear development and function has been demonstrated by studies in zebrafish and mice. Transcriptomic and proteomic approaches have been undertaken to identify miRNAs and their targets. Finally, it has been suggested that miRNAs may be used in the future in regeneration of inner ear hair cells and ultimately play a role in therapeutics.

## Introduction

While hints of the major regulatory force of microRNAs (miRNAs) appeared as early as 1989 (Ambros, 1989) and miRNAs as we know them today were first described in 2001 (Lagos-Quintana et al, 2001; Lau et al, 2001; Lee & Ambros, 2001), they did not appear on the scene in the inner ear until 2005, when miRNA expression was detected in the lateral line system and sensory organs of the zebrafish (Wienholds et al, 2005) (Table 1).

**Table 1:** Partial llst of ear miRNAs in zebrafish, mice and human tissue and their ear localization

*Danio rerio* miRNAs	Ear expression	Age	*Mus musculus* miRNAs	Ear expression	Age	*Homo sapiens* miRNAs	Ear expression
miR-96	Hair cells, otic neurons	Embryo	miR-96	Otic vesicle, cochlea-vestibular ganglion	E11.5	miR-96	
				Vestibular hair cells	E14.5		
				Hair cells	E17.5		
				Hair cells, spiral ganglia	P0		
				Spiral limbus, the inner sulcus	P4		
miR-182	Hair cells, otic neurons	Embryo	miR-182	Otic vesicle	E9.5	miR-182	
				Otic vesicle, cochlea-vestibular ganglion	E11.5		
				Vestibular hair cells	E14.5		
				hair cells	E17.5		
				Hair cells, spiral ganglia	P0		
				Hair cells	P4		
				Spiral limbus, the inner sulcus	P11-P15		
miR-183	Hair cells, otic neurons	Embryo	miR-183	Otic vesicle	E9.5	miR-183	
				Otic vesicle, cochlea-vestibular ganglion	E11.5		
				Vestibular hair cells	E14.5		
				Hair cells	E17.5		
				Hair cells, spiral ganglia	P0		
				Hair cells	P4		
				Spiral limbus, the inner sulcus	P11-P15		
miR-200a	Sensory epithelia	Embryo	miR-200a			miR-200a	
miR-200b			miR-200b	Sensory epithelia	P0	miR-200b	
miR-15a1	Utricular macula	Embryo	miR-15a	Hair and supporting cells, spiral ganglia	P0	miR-15a	
miR-18a	Utricular macula	Embryo	miR-18a	Hair and supporting cells, spiral ganglia	P0	miR-18a	
miR-30b			miR-30b	Hair and supporting cells, spiral ganglia	P0	miR-30b	
miR-99-1, -99-2			miR-99a	Hair and supporting cells, spiral ganglia	P0	miR-99a	
miR-199-1,-199-2,-199-3			miR-199a-2	Hair and supporting cells, spiral ganglia	P0	miR-199a-2	
miR-135b			miR-135b	Vestibular hair cells	P0	miR-135b	
miR-124			miR-124	Spiral and vestibular ganglia	P0	miR-124	
miR-21-1, -21-2			miR-21			miR-21	Cholesteatoma tissue, vestibular schwannoma

miRNA are small ∼23-nucleotide long non-coding RNAs that regulate gene expression. In mammals, a single miRNA can bind to hundreds of targets by complementation of the miRNA seed region to the 3′ UTR binding site of the target, thus reducing gene expression by translational suppression and mRNA destabilization. About one third of mammalian miRNA genes are located within introns of protein coding genes. Mostly oriented in the same orientation as their host mRNA, they use the same promoter for transcription, although some have an independent promoter (Guo et al, 2010).

Hearing loss is the most prevalent neurosensory disorder in humans. Congenital deafness affects approximately one in 500 newborns (National Institutes of Health, NIDCD, http://www.nidcd.nih.gov/health/statistics/hearing.html). The mammalian inner ear is comprised of two main organs, the cochlea that is responsible for hearing, and the vestibule that is responsible for perception of balance. The auditory capabilities of the cochlea are due to the organ on Corti. This organ, named after the Italian anatomist Corti (Editorial, 1952) is an extremely sensitive sensory epithelium, comprised of hair cells, specialized sensory cells with actin-rich stereocilia projections and non-sensory supporting cells (Frolenkov et al, 2004). miRNAs appeared in the mammalian inner ear field in 2006, with the description of miR-9 as a potential regulator of the COL9A1 short isoform (Sivakumaran et al, 2006). This was followed by a study of the miR-96, -182 and -183 cluster that transcribes polycistronically from one transcript. Their specific inner ear expression points to the importance of this triad in the inner ear (Weston et al, 2006). From that time on, the field exploded with a series of discoveries, the most compelling being the identification of a mutation responsible for deafness in two extended Spanish families. The causative mutation lay in the seed region of miR-96. This was the first example of a mutation found in a miRNA to lead to a Mendelian disease, and the first miR mutation shown to cause deafness (Mencia et al, 2009). miR-96 was also shown to cause deafness in an ENU-induced mouse and was extensively studied to understand the deafness-causing mechanism (Lewis et al, 2009). miRNAs were also described in other ear related diseases such as the role of miR-21 in human cholesteatoma growth and proliferation (Friedland et al, 2009) and in vestibular schwannomas (VS; Cioffi et al, 2010). miRNAs were found to regulate otitis media (OM) production and the inflammation process in cell lines derived from the middle ear (Song et al, 2011). Using new miRNA and target identification techniques, this fast growing field is constantly renewing, and more data of inner ear expressed miRNAs are being published. This review focuses on the latest findings of the involvement of miRNAs in human inner ear diseases, miRNA target identification and the mechanisms of miRNA function in the auditory system.

## Expression of miRNAs and implications for function

### Elucidating miRNA expression in the inner ear

The initial discovery of miRNAs in the inner ear was based on their expression (Table 1). Since then, a number of methods have been used to explore the temporal and spatial expression of miRNAs. Microarrays have generally been used for the study of gene expression profiles. They have been especially valuable for expression profiles of small RNAs, including miRNAs (Barad et al, 2004; Liu et al, 2004). This technique compares between the expression profiles of two samples, using hybridization of each sample to oligonucleotide-synthesized labelled probes. The first mammalian inner ear miRNAs were described following hybridization of whole cochlea at different time points to commercial miRNA microarrays (Weston et al, 2006). Affymetrix microarrays have been used for gene expression analysis between proliferating or non-proliferating chick auditory epithelia (Frucht et al, 2010). Thereafter, locked nucleic acid (LNA)-enhanced arrays were used, based on the nucleic acid analogue LNA (Petersen & Wengel, 2003) with high affinity to RNA (Elkan-Miller et al, 2011). This microarray platform, manufactured by Exiqon A/S (Denmark), is highly sensitive and specific, with both known and proprietary miRNAs.

Quantitative real-time polymerase chain reaction (qRT-PCR) is a rapid and sensitive method to quantify mature or precursor miRNAs. They are often used following identification of miRNA expression with microarrays, to both validate the finding and define the differential levels of expression accurately. This type of validation of miRNAs identified in the first mammalian microarray screen utilized SYBR Green fluorescence (Weston et al, 2006). This method was used further to examine changes in miRNA expression in cochlear progenitor cells (Hei et al, 2011). Other validations utilized the TaqMan system, which is based on stem and loop primers to convert RNA to cDNA, increasing the specificity and solving the problem of similarity of miRNA family members differing by only one nucleotide (Chen et al, 2005; Raymond et al, 2005). In a recent report comparing several expression profile methods for detection of miRNAs in a cancer cell line, there appeared to be a high correlation between the different technical methods (Sokilde et al, 2011).

*In situ* hybridization is widely used to characterize the spatial expression profile of miRNAs. The tissue, either in whole-mount form or in frozen or paraffin sections, is hybridized to an LNA™ modified detection probe, labelled with a digoxygenin (DIG) molecule, and followed by detection with an anti-alkaline phosphatase-conjugated (AP) antibody (Weston et al, 2006). The result is a purple staining where the miRNA is detected, after reacting with the alkaline phosphatase substrate. The most striking results using this technique have demonstrated that miRNAs have an extremely variable temporal and spatial expression pattern (Elkan-Miller et al, 2011; Friedman et al, 2009; Sacheli et al, 2009).

Due to the inaccessibility of human inner ear tissue and lack of relevant human inner ear cell lines, expression studies on miRNAs are limited to animals, with extrapolations to human function. For these reasons both the zebrafish and mouse are organisms of choice for evaluating ear miRNA function. Zebrafish are commonly used as a powerful tool to study mammalian systems and are a relevant model to study human disease (Driever & Fishman, 1996). These fish are small, easy to grow, translucent and are relatively easy to manipulate by gene silencing at the embryonic stage. The perception of hearing and balance in the zebrafish is performed by the lateral line and the mechanosensory organs (Nicolson, 2005). miRNA expression in the zebrafish inner ear was first demonstrated by mir-200a and mir-183 expression in the sensory epithelia (Wienholds et al, 2005). A conserved miRNA cluster, which includes miR-183, miR-182 and miR-96, was shown to be expressed in the zebrafish in the hair cells, otic neurons and other primary sensory cells. These findings were followed by a report that miR-15a-1 is found in neuromasts and throughout the inner ear and miR-18a is mainly in the utricular macula and nearby cells at 48 h post-fertilization (hpf) (Friedman et al, 2009). As some of these miRNAs were found to be restricted to the sensory organs, a role for miRNA regulation was implicated in this complex system.

**Glossary**

**Deafness**

A common neurosensory disorder that leads to partial or complete hearing loss.

**microRNA (miRNA)**

A ∼23-nucleotide long RNA that is not translated into protein and regulates gene expression by binding to the 3′UTR of a target mRNA.

**Cochlea**

The auditory portion of the inner ear labyrinth responsible for hearing.

**Vestibule**

A portion of the inner ear labyrinth responsible for balance.

**Hair cells**

Sensory receptor cells located in the cochlea and vestibule organs.

**Zebrafish**

The species *Danio rerio*, a tropical freshwater fish used as a vertebrate model organism.

**Cholesteatoma**

A benign keratinizing squamous epithelium growth in the middle ear.

**Vestibular schwannomas**

A benign vestibular nerve Schwann cell tumour.

**Otitis media**

An inflammation of the middle ear, mainly affecting children.

**Transcriptome**

The expression profile of messenger RNAs.

**Proteomics**

The large-scale study of proteins.

**Regeneration**

A process of renewal of dead or lost cells.

The mouse is considered to be the most relevant mammalian model to study mechanisms of deafness (Leibovici et al, 2008). Inner ear development in the mouse is an extensively researched field, and numerous deaf mouse mutants serving as models for human deafness have been studied (Frolenkov et al, 2004). The emerging interest with miRNA expression in the mouse inner ear and their connection to development and maturation of the inner ear began with a microarray expression analyses study throughout several developmental stages, from postnatal day zero (P0) to 5 weeks (Weston et al, 2006). A subset of miRNAs were then evaluated by Northern blot analysis using samples from the inner ear and other mouse organs, to assess whether there are tissue-specific miRNAs, with a special interest in the inner ear. These analyses revealed that the conserved cluster of mir-183, mir-182 and mir-96 have a restricted expression to the mouse inner ear, as compared to brain, heart and whole embryo expression. *In situ* hybridization revealed the unique expression pattern of mir-182, mir-183 and mir-96 in inner and outer hair cells of the cochlea, hair cells of the vestibular organs and spiral and vestibular ganglia. These three miRNAs were shown to be expressed in the same pattern (Weston et al, 2006), are transcribed in the same orientation and are predicted to be co-expressed (Wienholds et al, 2005). Another distinctive expression pattern was exhibited by miR-124, an abundantly expressed miRNA in the nervous system (Lagos-Quintana et al, 2002), which was found to be expressed in spiral and vestibular ganglia (Weston et al, 2006). This was the first report describing mammalian inner ear specific miRNAs, and their ubiquitous or restricted expression patterns.

**Figure 1:**
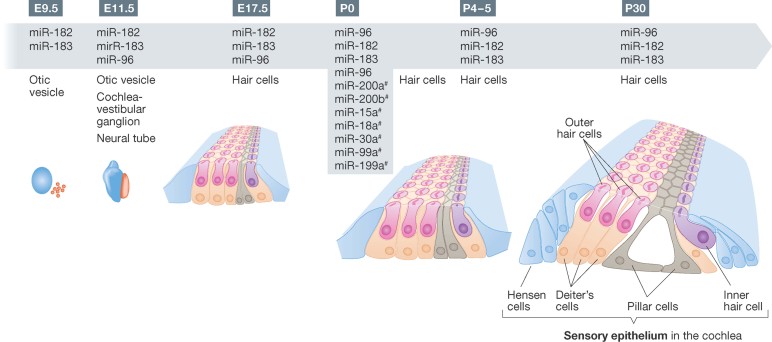
Timeline for miRNA expression during development and early postnatal stages of the inner ear for a subset of miRNAs The most well-studied miRNAs, mir-96, -182- and -183, were first detected in the otic vesicle at E9.5, progressed to the cochlea-vestibule ganglion and the neural tube at E11.5, and by E17.5 had strong expression in the cochlear hair cells. The expression continued to at least p30, by some reports. The expression of other miRNAs detected by *in situ* hybridization are shown as well. Those marked with # were only examined at the stage indicated.

In order to address the essential question of the effect of miRNAs throughout development, a study was conducted examining the expression pattern of the mir-183, mir-182 and mir-96 cluster (Sacheli et al, 2009). In this study, *in situ* hybridization analysis was performed at early time points, with miRNAs displaying distinctive developmental expression. The earliest observed expression was at embryonic day 9.5 (E9.5). miR-183 and miR-182, but not miR-96, were detected in the otic vesicle in the embryonic early inner ear. Later, at E11.5, all three miRNAs were detected in the otic vesicle, in the cochlea-vestibular ganglion, and the neural tube. The dramatic change in expression begins around E14.5, as at this time point this cluster's expression is limited to the vestibular hair cells. From E17.5 expression was detected in the hair cells of what will become the cochlea and the vestibular system, and were not detected in the supporting cells. By P0, miR-183, miR-182 and miR-96 were strongly expressed in hair cells of the cochlea and the vestibular system, and in the spiral ganglia. At P4 and on, there appeared to be another big shift in expression, as miR-96 was no longer observed in the cochlea or vestibule, but was detected in the spiral limbus and the inner sulcus. However, the expression of miR-183 and miR-182 continued in the hair cells, but ceased to be present in hair cells from P11-15, and was only found in the spiral limbus and the inner sulcus. Overall, these findings correlate with the maturation course of inner ear development and differentiation (Fig 1). The differentiation of the hair cells begins around E14.5, until around E17-E18, when the sensory epithelium exhibit hair cells throughout its length (Chen et al, 2002). The fact that expression of these miRNAs peak at the point of differentiation, and change from a general expression pattern in the sensory epithelium to restricted expression in the hair cells implicates these miRNAs in the development and maturation of the neonatal mouse inner ear.

Another study with an interest in the regulatory differences between the cochlea and the vestibular systems carried out a microarray scan of small RNAs from mouse P0 cochlea and vestibule (Friedman et al, 2009). Twenty-four miRNAs were found to be differentially expressed between the two tissues. An expression profile of a set of five miRNAs was evaluated using *in situ* hybridization. mir-15a, mir-18a, mir-30b, mir-99a and mir-199a were found in different and distinct regions of the mouse P0 cochlea and vestibule, including hair and supporting cells, the spiral ganglia and other cell types. The different expression patterns demonstrated that miRNA expression in the inner ear is not restricted to one cell type or developmental stage, and their expression is spread and is associated with the developmental changes that occur during the course of time (Fig 1).

### miRNAs are involved in formation of the inner ear

Once it was established that miRNAs are expressed extensively throughout inner ear and during development, the involvement of *Dicer1* in the formation of the inner ear was addressed as well. Type III ribonuclease *Dicer1* is essential to the processing of mature miRNAs from their pre-miRNA form (Bernstein et al, 2001). Mice lacking the *Dicer1* enzyme, created by a knock-out (KO) gene deletion technique, die during embryogenesis, as observed as early as E7.5, probably due to the inability of the embryo to produce functional mature miRNAs that may regulate developmental processes (Bernstein et al, 2003).

To overcome this early death, conditional knock-outs were created using the *Cre* transgene (Harfe et al, 2005). The first study investigating the role of *Dicer1* in the development of the inner ear was conducted using *Pax2-Cre* transgenic mice (Soukup et al, 2009). The conditional *Dicer1* deletion in the *Pax2-Cre* mice allows for *Dicer1* deletion in the inner ear, kidney and midbrain upon crossing to the *Dicer1-loxP* mice. The dramatic influence of the loss of *Dicer1* was demonstrated by the mice dying by E18.5. The inner ear malformations were extensive. By E17.5 there was a major truncation of the inner ear structure, and the cochlea was missing the coiled shape. Scanning electron microscopy (SEM) revealed that cochlear hair cells exhibited abnormal stereocilia organization. A different conditional *Dicer1* KO model used *Cre* expression downstream to the *Pou4f3* promoter in an attempt to create viable mice (*Dicer*-PCKO) (Friedman et al, 2009). The *Pou4f3* promoter was chosen for the *Dicer1* conditional KO mouse model as it is expressed in the hair cells. Restricting the expression of *Cre* recombinase in these cells resulted in the removal of the *Dicer1-LoxP* sites from the hair cells, leading to depletion of *Dicer1* expression in hair cells. The majority of defects in the auditory system were observed in the adult mice. *Dicer*-PCKO p38 mice were deaf, as was demonstrated by auditory brainstem response (ABR), and also showed a mild circling vestibular phenotype. Most of the cochlear hair cells were misshaped and did not express myosin VI, a hair cell marker and SEM revealed that many of the hair cells either lost their stereocilia or had abnormal shapes. These results demonstrate a more profound inner ear malformation achieved by interrupting the miRNA maturation process. The next model used a *Foxg1-Cre* mediated knock out of *Dicer1* (Kersigo et al, 2011). The effect on the inner ear was significant, with reduction in the inner ear size observed at E14.5, followed by a loss of canal formation and a loss of the coiled cochlea structure by E18.5. These models indicate that normal development of the functioning inner ear is strongly dependent on normal miRNA maturation. Disruption of the mature miRNA production caused not only structural defects, but a loss of auditory and vestibular function.

The last model used so far to investigate the influence of *Dicer1* on inner ear development is a *Atoh1-Cre* conditional *Dicer1* knockout. *Atoh1* is expressed in all hair cells during the embryonic stage, but is not restricted to the inner ear. Mice died around the age of 4 weeks, after exhibiting ataxia and seizures. miR-183 was completely depleted in the hair cells of the mutant mice, but not in spiral ganglia. In addition, expression of miRNAs was observed in other inner ear components, but not in hair cells, indicating that this model specifically suppresses the expression of miRNA in hair cells (Weston et al, 2011). These findings suggest that miRNAs are crucial for inner ear development, and are involved in the formation, morphogenesis and neurosensory processes that create the functional auditory organ.

### Approaches to miRNA target identification

In order to understand how miRNAs function in cells, their targets must be identified. miRNAs destabilize target mRNAs or inhibit protein translation, leading to decreased protein levels (Guo et al, 2010). These targets may be important cell regulators and their decrease, even by a small amount, can change the course of the cell. Target prediction has been a challenge since the discovery of miRNAs; however, algorithms to find real functional targets are improving. There are a number of publicly available target prediction programs, including but not limited to TargetScan and miRanda, most of which work by slightly different algorithms, although they all rely on seed pairing of miRNA-target recognition. They search for the complementation of the miRNA 5′ seed region nucleotide (nt) 2–7 to the 3′UTR of the target, and the evolutionary conservation of the 7 nt in 3′UTR of targets, matching the miRNA seed region (Bartel, 2009). The significant shortcoming of these computational prediction programs is the large amount of false positive noise they produce. For example, although a miRNA-target pair could be found using such a method, both the miRNA and target are not necessarily expressed in the studied tissue and thus do not interact *in vivo*. In such a case, the prediction is not relevant. This conclusion emphasizes the importance of biological validation of each computationally predicted miRNA-target pair. Identification of new targets for miRNAs expressed in the inner ear has been demonstrated in a few studies (Elkan-Miller et al, 2011; Hertzano et al, 2011).

A computational analysis using the miRanda target prediction tool was conducted to produce a list of more than 100 putative targets for miR-96 (Lewis et al, 2009). The list was annotated and 13 potential targets were then examined biologically using a luciferase assay (Lewis et al, 2009). Five of the tested targets were validated biologically as miR-96 targets: *Aqp5*, *Celsr2*, *Myrip*, *Odf2* and *Ryk*. Using the miR-96 mouse mutant diminuendo, a different target identification approach was used. Microarray gene expression analysis was done to compare the differentially expressed set of mRNAs from the mutant diminuendo mouse and a wild type (WT) control. After qRT-PCR confirmation, five genes were found to be down-regulated in the mutant: *Slc26a5* (prestin), *Ocm* (oncomodulin), *Pitpnm1*, *Gfi1* and *Ptprq*. These genes do not appear to be direct targets of miR-96, suggesting that their down-regulation is a downstream effect.

**Figure 2:**
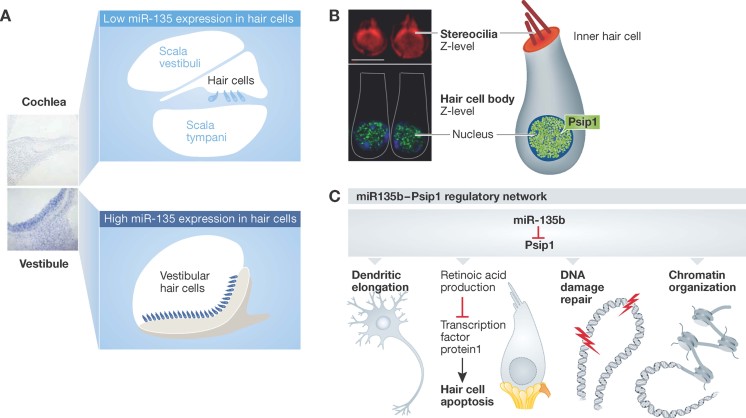
miRNA target identification Computational and experimental approaches have been taken to identify target of miRNA in the inner ear. An example of such a miR-target pair is miR-135 and Psip, PC4- and SF-2 interacting protein/Ledgf (Elkan-Miller et al, 2011), which has been implicated in transcriptional regulation of stress-related genes, having an anti-apoptotic effect, involved in mRNA splicing, cell survival and is part of a fusion gene in leukaemia. miR-135 is reduced in the cochlear hair cells, while its expression is high in vestibular hair cells.Psip1, one of its targets, is expressed in the nucleus of the hair cells.The pathways shown demonstrate potential inner ear functional pathways implicated in the miR135b-Psip regulatory network. Psip1 is a transcriptional regulator that plays a role in retinoic acid production (Fatma et al, 2004). Retinoic acid is crucial for hair cell development (Raz & Kelley, 1999). This model suggests that miR-135b regulation of Psip1 plays a role in hair cell development and survival.

Another study used a combination of experimental data and computational analysis to predict functional targets for miRNAs in the inner ear (Elkan-Miller et al, 2011). miRNAs were identified after applying a transcriptomic and proteomic expression profiling to search for differentially expressed miRNAs between the cochlea and vestibular P2 mouse sensory epithelia. Functional targets were then searched for by using the FAME (Functional Assignment of miRNA via Enrichment) algorithm (Ulitsky et al, 2010). FAME evaluates enrichment and depletion of potential targets, found by TargetScan, in a data set of up or down-regulated mRNAs and proteins. In this study, miR-135b was found in LNA arrays to be up-regulated in the vestibule, while its targets were enriched in a protein dataset and down-regulated in the vestibule. *In situ* hybridization confirmed the differential expression of miR-135b in vestibular hair cells as compared to cochlear hair cells at P0. The analysis predicted PSIP1-P75 as one of potential targets of miR-135b. Psip, PC4- and SF-2 interacting protein/Ledgf is a known transcriptional activator implicated to regulate stress-related genes, has an anti-apoptotic effect, is involved in mRNA splicing, cell survival and is part of a fusion gene in leukaemia (Sutherland et al, 2006). Both qRT-PCR and protein expression was consistent with the FAME predictions, as PSIP1-P75 was down-regulated in the vestibule, as compared to the cochlea. Finally, the miRNA-target interaction was verified by both an RNA interference (RNAi) silencing technique and a luciferase reporter assay. This example illustrates a target identification approach, combining both *in silico* analysis and experimental data to be able to predict functionally relevant targets. Furthermore, the pathways involving PSIP1-P75 are known in other tissues and can provide guidelines for determining the function of this miRNA-target pair in the inner ear (Fig 2).

The final example of a complex approach to search for functional targets was demonstrated by a targeted search of inner ear regulators of cell fate, including miRNA targets and transcription factors, using a transcriptome cell type-specific study (Hertzano et al, 2011). Flow cytometry of vestibular and cochlear tissue using CD326, CD34 and CD49f markers allowed separation of epithelial (sensory and nonsensory), neuronal and vascular cell populations to identify differentially expressed transcripts between these eight distinct cell populations. Using this data, miRNA families whose potential targets were differentially expressed in a specific cell type, in the opposite direction of the miRNA expression, were coupled. The prediction identified the miR-200b family, with potential targets down-regulated in sensory epithelial cells at P0. miR-200b expression was detected by *in situ* hybridization in all sensory epithelial cells of the inner ear. In order to identify other regulatory factors in the inner ear, a search was made for *cis*-regulatory elements over expressed in promoters of sensory epithelial cells in the inner ear. This bioinformatics technique revealed the transcription factors ZEB1 and ZEB2 as regulators of genes expressed in the inner ear sensory cells. It has been previously shown that miR-200b directly targets ZEB1 and ZEB2 (Burk et al, 2008), linking the regulatory factors to one another. Finally, using a cell type-specific approach, they demonstrated marked mis-expression of genes suggested to be regulated by Zeb1 in a mouse mutant with a mutation in the Zeb1 transcription factor. The results of this study uncovered a complex regulatory network of miRNAs, targets and transcriptional repressors.

## Clinical implications of inner ear miRNAs

### Mutations in miRNAs lead to human deafness

The importance of miRNAs in inner ear development and their involvement in the functional hearing process has been established by expression studies and animal models. The crucial nature of miRNAs in auditory function was made evident by the discovery that mutations in the miR-96 gene are the underlying cause for non-syndromic hearing loss in two large human families (Mencia et al, 2009). Two mutations in the seed region of miR-96, +13 G > A and +14 C > A, were discovered in unrelated Spanish families exhibiting autosomal dominant progressive hearing loss. This finding suggests that the deafness is a result of the loss of the target recognition site and the gain of new targets, both consequences of the change in the miR-96 seed region. The identification of the mutations in the miR-96 gene was the first report in humans of a miRNA mutation causing a Mendelian disease. Since this report, several studies have focused on the mir-96, mir-182 and mir-183 genes as a source for more deafness mutations. However, no mutations were found in these genes in populations of Americans and Iranians with autosomal dominant and recessive non-syndromic hearing loss, respectively, and in Iranian autosomal recessive non-syndromic hearing loss families (Hildebrand et al, 2010) suggesting mutations in this cluster are not a common cause of deafness. Following this study, a new mutation resulting in hearing loss was discovered in the miR-96 gene in an Italian family, resulting in hearing loss (Solda et al, 2012). Unlike the first miR-96 mutations, this mutation (+57 T > C) is not located in the seed region. The mutation was shown to interfere with the miR-96 pre-miRNA secondary structure, changing the miR-96-3p sequence and lowering the number of copies of the mature miR-96. The fact that mutations in miRNAs can cause hearing loss demonstrates unequivocally the essential role they have in auditory function, and furthermore, can help define the mechanisms by which a single miRNA mutation can result in deafness.

### Model systems address mechanisms of the human mutations

Parallel to the discovery of the human miR-96 mutation, a mutation was found in miR-96 in an *N*-ethyl-*N*-nitrosourea (ENU)-induced deaf mouse (Lewis et al, 2009). This mouse, named diminuendo (*Dmdo*), exhibits progressive autosomal dominant hearing loss, similar to the phenotype in humans. The hearing loss and balance phenotype was more severe in the homozygote, and SEM revealed stereocilia malformations in both the heterozygote and homozygote mice. A point mutation was discovered, after chromosomal mapping to chromosome 6, in the mir-96 seed region (+15 A > T), a region fully conserved between species. The mutation did not disturb the formation of the mature miR-96 in the mutant and therefore the search for the mechanism was shifted to target recognition, as described above. However, neither the identified targets nor the down-regulated genes could be linked to the phenotype. Thus, it appears that since a single miRNA can regulate hundreds of targets, such a mutation in the seed region target-binding site can lead to a cascade effect, resulting in many secondary effects that eventually lead to deafness. Further analysis of the miR-96 mouse mutant found that hair cell maturation stops at P0 (Kuhn et al, 2011). The biophysical development of the hair cells was abnormal, as the inner hair cells were lacking the typical K+ current. This study suggests that miR-96 is required for embryonic hair cell development and maturation.

In a different model system, zebrafish were used to understand the role of the miR-96, miR-182 and miR-183 cluster in inner ear development (Li et al, 2010). The cluster was over expressed in zebrafish by injecting embryos with double-strand miRNAs. Embryos with miR-182 and miR-96 over-expression exhibited body malformations and produced ectopic hair cells. A knockdown of each of the miRNAs in the cluster using morpholino oligonucleotides showed a decrease in hair cell number. Over-expression of miR-182 in a miR-96 knockdown embryo demonstrated a partial rescue effect, and the number of hair cells was increased, as compared to the knockdown. These findings suggest that the cluster plays a role in hair cell formation and development, and that there is some effect of redundancy between these miRNA family members.

### Other human disease phenotypes in the ear

**Figure 3:**
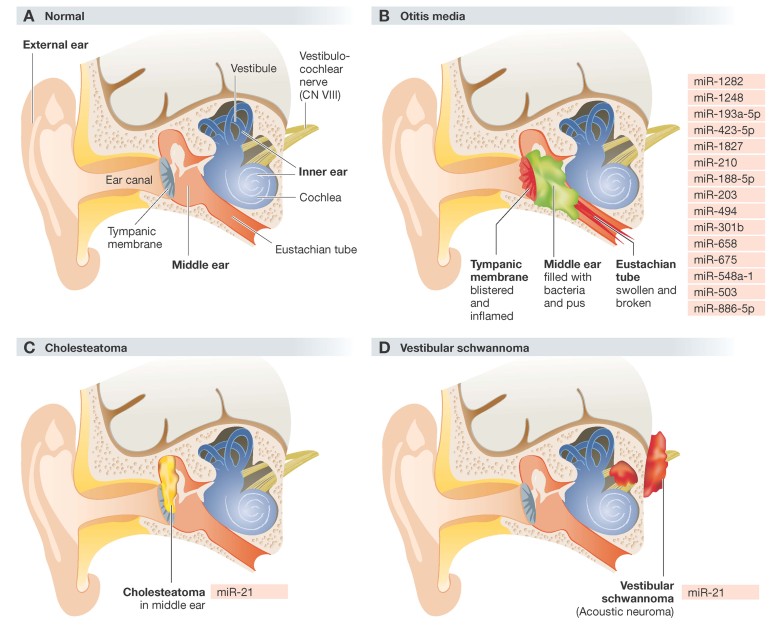
miRNA involvement in three human ear disease phenotypes The human ear is composed of the external, middle and inner ear.Otitis media (OM) is a middle ear inflammation. Inflammation was induced in HMEEC cells and their miRNA levels were examined by microarray analysis, revealing up- and down-regulation of a number of miRNAs involved in inflammation and growth (Song et al, 2011).A study examining cholesteatomas revealed upregulation of miR-21, leading to a model explaining the potential growth of this benign yet potentially harmful growth in the middle ear or the mastoid bone (Friedland et al, 2009).Vestibular schwannomas have also been found to contain increased levels of miR-21 (Cioffi et al, 2010).

miRNAs were discovered to regulate other ear-related human clinical features. One of the studies discovered a regulatory role of miR-21 in human cholesteatoma growth and proliferation (Friedland et al, 2009; Fig 3). Cholesteatomas are a benign expending growth, derived from the epidermis in the middle ear or the mastoid bone. It is often recurrent and may lead to the disruption of the temporal bone and to sensorineural hearing loss, among other symptoms. The molecular mechanisms leading to this growth are not well understood, and it has been suggested that miRNAs are potential molecular regulators in this pathology. In this study, samples of cholesteatomas and normal skin were taken from patients and analysed using qRT-PCR to detect up-regulated miRNAs. From a set of suspected miRNAs previously linked to proliferation and growth in other models, miR-21 was found to be up-regulated in cholesteatoma samples, as compared to normal skin. A regulatory model was suggested, based on known miR-21 targets, involving infection as the first stimulation, followed by factors that activate miR-21 transcription, which then suppresses PTEN and PDCD-4 (known tumour suppressor genes) and leads to cholesteatoma growth.

In a different study, miR-21 was found to be over-expressed in VS nerve tissue, as compared to a normal tissue (Cioffi et al, 2010; Fig 3). VS are benign vestibular nerve Schwann cell tumours. VS can cause vertigo, tinnitus, hearing loss and other symptoms. PTEN, a known miR-21 target, was found to decrease in protein levels, but not in the mRNA levels of VS samples. miR-21 was knocked down in primary VS cultures using anti-miR-21 oligonucleotides, which increased cell proliferation and survival.

Another human disease that has been addressed in miRNA research is OM (Song et al, 2011; Fig 3). OM is an inflammation of the middle ear. This condition is fairly common in children and left untreated, can lead to hearing impairment. In a study conducted using human middle ear epithelial cells (HMEECs) as a model for OM to identify miRNAs induced during inflammation, lipopolysaccharide (LPS)-treated and untreated cells were compared after microarray analysis. Five and ten miRNAs were found to be up-regulated and down-regulated, respectively. Many of the predicted targets of the up-regulated miRNAs are involved in acute inflammation response, development and cell growth regulation, as annotated with DAVID, a bioinformatics resource that provides functional annotation of genes. Among the down-regulated miRNA targets, functional targets are involved in development, cell communication, endocytosis, cell differentiation, IκB and NFκB cascade, complement activity, cell adhesion and the innate immune response. The authors suggested that this study implicates miRNAs in the immune response and OM formation.

These examples demonstrate a broad spectrum of miRNAs in human clinical features and diseases of the ear. Furthermore, it emphasizes the significance of miRNA studies to help better understand complicated mechanisms leading to human disease, particularly in the auditory and vestibular organs.

## Future directions in miRNA research

### Involvement in regeneration

Following the discovery of the numerous processes in which miRNAs are involved in the inner ear, it has become intriguing whether miRNAs are involved in a central focus of inner ear research, hair cell regeneration. In mammals, once the auditory hair cells are fully formed, regeneration does not occur after damage, especially in the cochlear hair cells (Stone & Cotanche, 2007). Nevertheless, in birds, reptiles, amphibian and fish, lost hair cells can be replaced by new ones, regenerating from supporting cells. This fact has been the basis for many studies, focusing on identifying the biological processes that are responsible for such regeneration, including miRNAs. In a study that investigated the role of miRNAs in transdifferentiation, a miRNA microarray analysis was performed using regenerating lens and inner ear hair cell tissues extracted from the adult newt. The labyrinth that includes the auditory and vestibular sensory epithelia was isolated and cultured *in vitro*. Hair cell death was induced with use of aminoglycosides and RNA samples from the cultured tissue were taken at a few time points, until regeneration was observed 18 days after treatment. Microarray analysis revealed that the *let-7* family, particularly let-7a, -7b, -7c, -7d, -7e, -7f and -7g, expression was reduced, both in hair cells and lens differentiation. Since the let-7 family is known to regulate terminal differentiation, their down-regulation suggests a potential role in dedifferentiation, thus regulating targets involved in regeneration (Tsonis et al, 2007).

In mammals, although the auditory hair cells do not regenerate, the vestibular sensory epithelium has a limited ability to regenerate (Kawamoto et al, 2009). The differences in the regulation between the cochlea and the vestibule are being studied, in order to determine where miRNA regulation is involved in this difference in regenerative ability. In the search for a differentially expressed miRNAs between the cochlea and the vestibule, a microarray analysis of P0 mouse cochlea and vestibule was performed (Friedman et al, 2009). Twenty-four miRNAs showed a differential expression. Despite the relatively moderate differences (15–40%), miRNAs that had the highest differential expression were let-7a, -7b, miR-220, -423, -190, -204,-24, -195 and miR-125b. These results suggest that miRNAs may be involved in regulating the different characteristics between the cochlea and the vestibule.

In contrast to mammals, the hair cells of the avian auditory epithelium have the capacity to regenerate upon damage (Brignull et al, 2009). In a search for genes involved in regeneration in the chicken, gene expression was studied using microarray analysis after applying forskolin treatment, which causes proliferation of supporting cells, followed by appearance of new hair cells on chicken basilar papilla (Frucht et al, 2010). The down-regulated genes between the treated tissue and the control represent a set of targets of miRNAs that are involved in the regeneration process. Using these two sets of tissues, a gene set enrichment analysis (GSEA) was performed. A few miRNAs were identified in the enrichment analysis, such as miR-181a, miR114, miR-200a and miR-27, suspected as being active miRNAs in the regeneration. miR-181a was further studied and was over expressed in chick basilar papilla explants. Following BrdU incorporation, explants transfected with the miR-181a showed higher levels of proliferation as compared to a control. For additional investigation of the role of miR-181a in proliferation, basilar papilla was treated with forskolin and later transfected with anti-miR-181a and treated with forskolin again. The basilar papillae transfected with both forskolin and anti-miR-181a had less proliferating cells, as compared to those only treated with forskolin and a control. These results suggest that miR-181a can encourage proliferation in both quiescent and proliferating chick basilar papilla. Zebrafish provides another example of the involvement of miRNAs in regeneration, as over-expression of miR-182 and miR-96 resulted in production of ectopic hair cells (Li et al, 2010). These studies have led to the idea that miRNAs may have a promising therapeutic aspect, since miRNAs have been implicated as both the active agent promoting regeneration, and as the regulatory mediator helping to uncover downstream targets and transcription factors involved in regeneration.

### Deep sequencing to identify new miRNAs

New developing technologies have brought with them remarkable opportunities for the discovery of new miRNAs. Next generation sequencing, or massively parallel sequencing (MPS), of DNA and RNA molecules has altered the speed and breadth of sequencing available in the past. While MPS began as a method to parallel sequence genomes on a wide scale, as the technology advanced, it was used to identify non-coding RNAs as well, including miRNAs (Berezikov et al, 2006; Mardis, 2008). Deep sequencing allows for the discovery of new miRNAs because of their unique structure and furthermore, can serve as a quantitative tool to directly measure miRNA abundance.

Thus far, the only study using this technology in the inner ear field has been for exploration of the changes in miRNA expression in the auditory forebrain of the zebra finch, after being exposed to song (Gunaratne et al, 2011). Deep sequencing conducted on RNA derived from auditory forebrain revealed 34 novel miRNAs specific to the zebra finch genome. This example demonstrates that miRNA expression can change through behaviour and neuronal response, and can be detected by deep sequencing. As prices of deep sequencing decrease, and the technology becomes more available worldwide, we should expect that the use of this sequencing technology to identify new miRNAs in a variety of tissues, during developmental changes or expression in a disease, will be utilized more widely.

## Conclusions

miRNA involvement in the development and maturation of the inner ear and the auditory mechanism has been demonstrated numerous times. The most relevant to inner ear disease is the mutations in the seed region of miR-96, causing deafness in both humans and mice. This remarkable finding initiated a new paradigm in the deafness gene hunt. This was the first time that a single mutation in a miRNA was found to lead to deafness. The implication that a single mutation in a miRNA can lead to deafness is not trivial, since it is not a direct result of the mutation, but rather, a result of many small cumulative events, caused by both the loss of inhibition on the miR-96 targets, and gain of function of the targets now recognized by the new mutated seed region. This complex disease-causing mechanism had led us to believe that there is much more to miRNA involvement in disease than previously known. As new deep sequencing technologies are becoming more readily available, the miRNA field will rapidly grow and expand, identifying new miRNAs regulating important inner ear functions. Many diseases may be demonstrated to be affected by miRNA mutations, opening a vast potential for a future role for miRNAs as therapeutic agents.

**Pending issues**

**Lack of human RNA tissue from the inner ear**: Unlike miRNA research in the cancer field, where much of the work is focused on tissues and tumours derived from human patients, research in the deafness field does not base itself on human tissue. RNA from humans is almost impossible to obtain. Despite the genetic resemblance between humans and mice, miRNA targets may be different between the two, providing a difficult basis for development of therapeutics for human deafness. This aspect must be reconciled with when making extrapolations from miRNA experiments using RNA derived from mice.

**Regeneration**: Regeneration of damaged hair cells does not occur in the mammalian cochlea. miRNA involvement in hair cell regeneration in reptiles and birds must be further studied, with the hope of identifying factors that promote regeneration in mammals.

**Treatment**: Treatment by gene therapy for diseases caused by miRNA mutations is still the ‘holy grail’ of miRNA disease research. A study of the pathogenesis caused by a mutation in a single miRNA, such as miR-96, is a promising candidate for gene therapy studies. Finding methods to reduce or induce miRNA expression in the inner ear, for fine-tuning of target expression to influence factors involved in hearing loss, is an ongoing goal in the field.

## For more information

National Institutes of Health, NIDCD: http://www.nidcd.nih.gov/health/statistics/hearing.html

Hereditary Hearing Loss Homepage: http://hereditaryhearingloss.org/

miRBase, microRNA database: http://www.mirbase.org/

TargetScan: http://www.targetscan.org/

miRanda: http://www.microrna.org/microrna/home.do

FAME (Functional Assignment of MiRNA via Enrichment): http://acgt.cs.tau.ac.il/fame/

DAVID: http://david.abcc.ncifcrf.gov/
